# Crystal structure of a hypothetical protein from *Giardia lamblia*

**DOI:** 10.1107/S2053230X21013595

**Published:** 2022-01-28

**Authors:** Dylan K. Beard, Seonna Bristol, Kayla Cosby, Amber Davis, Courtney Manning, Lionel Perry, Lauren Snapp, Arian Toy, Kayla Wheeler, Jeremy Young, Bart Staker, Tracy L. Arakaki, Jan Abendroth, Sandhya Subrahamanian, Thomas E. Edwards, Peter J. Myler, Oluwatoyin A. Asojo

**Affiliations:** a Department of Chemistry and Biochemistry, Hampton University, 100 William R. Harvey Way, Hampton, VA 23668, USA; b Seattle Structural Genomics Center for Infectious Disease (SSGCID), Seattle, Washington, USA; c Center for Infectious Disease Research, formerly Seattle Biomedical Research Institute, 307 Westlake Avenue North Suite 500, Seattle, WA 98109, USA; d Labcorp Drug Development Inc., Princeton, NJ 08540, USA

**Keywords:** giardiasis, SSGCID, infectious diseases, travelers’ diarrhea, undergraduate education and training, structural genomics

## Abstract

Giardiasis is the most prevalent diarrheal disease globally and affects humans and animals. It is a significant problem in developing countries, the number one cause of travelers’ diarrhea and affects children and immunocompromised individuals, especially HIV-infected individuals. Giardiasis is treated with antibiotics (tinidazole and metronidazole) that are also used for other infections such as trichomoniasis. The ongoing search for new therapeutics for giardiasis includes characterizing the structure and function of proteins from the causative protozoan *Giardia lamblia*. These proteins include hypothetical proteins that share 30% sequence identity or less with proteins of known structure. Here, the atomic resolution structure of a 15.6 kDa protein was determined by molecular replacement. The structure has the two-layer αβ-sandwich topology observed in the prototypical endoribonucleases L-PSPs (liver perchloric acid-soluble proteins) with conserved allosteric active sites containing small molecules from the crystallization solution. This article is an educational collaboration between Hampton University and the Seattle Structural Genomics Center for Infectious Disease.

## Abstract 2

The 1.35 Å resolution crystal structure of a 15.6 kDa hypothetical protein from the parasite *Giardia lamblia* was determined as part of structural genomic studies to assign possible functions to hypothetical proteins from infectious agents. The structure has a prototypical endoribonuclease L-PSP (liver perchloric acid-soluble protein) topology with conserved allosteric active-site residues despite lacking any appreciable sequence identity to other members of this superfamily.

## 1. Introduction

The flagellated protozoa *Giardia lamblia* is the most commonly identified intestinal parasite globally, causing giardiasis, otherwise known as travelers’ diarrhea (Daniels *et al.*, 2015 ▸; Escobedo *et al.*, 2015 ▸). Giardiasis is a zoonotic infection, and *Giardia* species have been isolated from the stools of vertebrates, including mammals, amphibians and birds (Thompson, 2013 ▸). *Giardia* is an endemic neglected tropical disease, and outbreaks of giardiasis from contaminated water or food sources are common in developing countries because of poor sanitation (McIntyre *et al.*, 2014 ▸). It only takes ∼10 *Giardia* cysts to cause infection, and in developed countries giardiasis is more common among children and hospital patients, especially immunocompromised individuals and institutionalized patients (Huang & White, 2006 ▸). The current standard treatment for giardiasis is antibiotic therapy using tinidazole and metronidazole (Lobovská & Nohýnková, 2003 ▸). Characterizing the structures and functions of *G. lamblia* proteins is the first step towards identifying new therapeutics for giardiasis.

**Figure 1:**
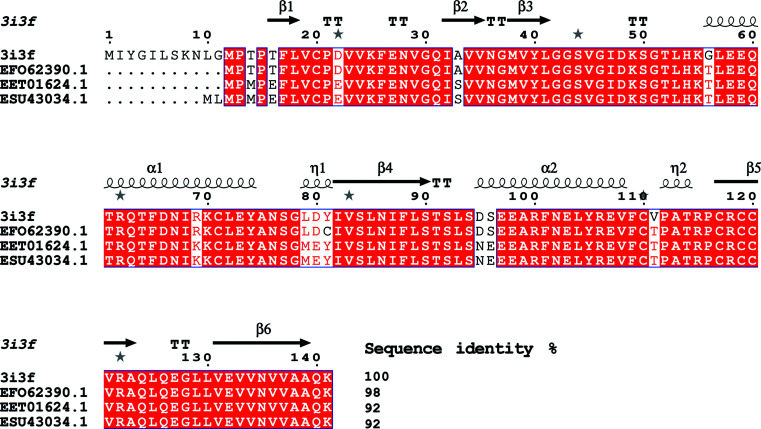
Structural and primary-sequence alignment of the hypothetical protein from *G. lamblia* (GilaA.00312.a) with EFO62390.1, the hypothetical protein GLP15_656 from *G. lamblia* P15, EET01624.1, the hypothetical protein GL50581_1093 from *G. intestinalis* ATCC 50581, and ESU43034.1, a putative YjgF/YER057c/UK114 family protein from *G. intestinalis*. The secondary-structure elements shown are α-helices (α), 3_10_-helices (η), β-strands (β) and β-turns (TT). Identical residues are shown in white on a red background and conserved residues are shown in red. This figure was generated using *ESPript* (Gouet *et al.*, 1999 ▸, 2003 ▸).

*G. lamblia* is one of the organisms selected by the Seattle Structural Genomics Center for Infectious Disease (SSGCID) for high-throughput structural studies, and hypothetical proteins have been identified with limited sequence similarity to proteins of known function. One of these hypothetical proteins is a 141-amino-acid protein (UniProt ID A8BD71, XP_001707732.1). This protein shares over 30% sequence identity and 50% coverage with only two unique proteins in the Protein Data Bank. One of these proteins is a putative endonuclease from *Entamoeba histolytica* (PDB entries 3mqw, 3m1x and 3m4s; 36% sequence identity and 56% coverage; Seattle Structural Genomics Center for Infectious Disease, unpublished work). The other comprises the amino-terminal residues 13–121 of *Saccharomyces cerevisiae* mitochondrial matrix protein Mmf1 (PDB entry 3quw), with 30% sequence identity and 77% coverage (Pu *et al.*, 2011 ▸). A *BLAST* search against all redundant *Giardia* sequences reveals three proteins that share appreciable sequence similarity with this hypo­thetical protein: EFO62390.1, the hypothetical protein GLP15_656 from *G. lamblia* P15, EET01624.1, the hypo­thetical protein GL50581_1093 from *G. intestinalis* ATCC 50581, and ESU43034.1, a putative YjgF/YER057c/UK114 family protein from *G. intestinalis* (Fig. 1 ▸). Here, we present the atomic resolution crystal structure of this hypothetical protein as a first step towards clarifying its possible functions.

## 2. Materials and methods

### 2.1. Macromolecule production

The protein was cloned, expressed and purified following standard protocols of the Seattle Structural Genomics Center for Infectious Disease (SSGCID; Bryan *et al.*, 2011 ▸; Choi *et al.*, 2011 ▸; Serbzhinskiy *et al.*, 2015 ▸). Briefly, genomic DNA from *G. lamblia* GL50803_14299 was provided by Dr Ethan Merritt, University of Washington. DNA encoding amino acids 1–141 (UniProt A8BD71) of *G. lamblia* GL50803_14299 was PCR-amplified from genomic DNA using the primers shown in Table 1 ▸. The PCR product was cloned into expression vector pAVA0421 (Choi *et al.*, 2011 ▸) by ligation-independent cloning (LIC; Aslanidis & de Jong, 1990 ▸). The final expression vector includes a cleavable 6×His fusion tag followed by the human rhinovirus 3C protease-cleavage sequence (MAHHHHHHMGTLEAQTQGPGS-ORF). The underlined glutamine (Q) and glycine (G) residues denote the 3C cleavage site. Plasmid DNA was transformed into chemically competent *Escherichia coli* BL21(DE3)R3 Rosetta cells. The cells were tested for expression and 2 l of culture was grown using auto-induction medium (Studier, 2005 ▸) in a LEX Bioreactor (Epiphyte Three Inc.). The expression clone was assigned the SSGCID target identifier GilaA.00312.a.

Both the expression clone and purified protein are available at https://www.ssgcid.org/available-materials/.

The recombinant protein was purified using a four-step protocol consisting of an Ni^2+^-affinity chromatography (IMAC) step, cleavage of the N-terminal histidine tag with 3C protease, reverse capture with a second Ni^2+^-affinity chromatography column and size-exclusion chromatography (SEC). All chromatography runs were performed on an ÄKTA­purifier 10 (GE) using automated IMAC and SEC programs according to previously described procedures (Bryan *et al.*, 2011 ▸). The final SEC was performed on a HiLoad 26/600 Superdex 75 column (GE Healthcare) using a mobile phase consisting of 500 m*M* NaCl, 25 m*M* HEPES, 5% glycerol, 0.025% azide, 2 m*M* DTT pH 7.0. Peak fractions were pooled and analyzed using SDS–PAGE. The peak fractions were concentrated to 30.5 mg ml^−1^ using an Amicon purification system (Millipore). Aliquots of 200 µl were flash-frozen in liquid nitrogen and stored at −80°C until use for crystallization.

### 2.2. Crystallization

Crystals were grown following established crystallization approaches at the SSGCID. Briefly, recombinant GilaA.00312.a was diluted to 13.46 mg ml^−1^. Protein concentration was assessed using the OD_280_ with a molar extinction coefficient of 7450 *M* ^−1^ cm^−1^. Single crystals were obtained by vapor diffusion in sitting drops using equal volumes of protein solution and precipitant solution equilibrated against a reservoir containing precipitant solution (Table 2 ▸).

### 2.3. Data collection and processing

Data collection and processing were performed using established protocols at the SSGCID. Specifically, a single crystal was transferred into cryosolution (buffer solution plus 20% ethylene glycol), flash-cooled in liquid nitrogen and transferred into a puck for data collection on APS beamline 21-ID-F. Data were processed using *XDS*/*XSCALE* (Kabsch, 2010 ▸). Additional data-collection information is provided in Table 3 ▸.

### 2.4. Structure solution and refinement

The structure was solved by molecular replacement using *MOLREP* (Lebedev *et al.*, 2008 ▸; Vagin & Teplyakov, 2010 ▸) with the structure of yeast mitochondrial matrix factor 1 (PDB entry 1jd1; Deaconescu *et al.*, 2002 ▸) as the search model. Initial refinement was carried out with *REFMAC* (Murshudov *et al.*, 2011 ▸) with TLS, with manual refinement in *Coot* (Emsley & Cowtan, 2004 ▸; Emsley *et al.*, 2010 ▸). The structure quality was checked by *MolProbity* (Headd *et al.*, 2009 ▸) and the resulting structure-refinement data are provided in Table 4 ▸.

## 3. Results and discussion

**Figure 2:**
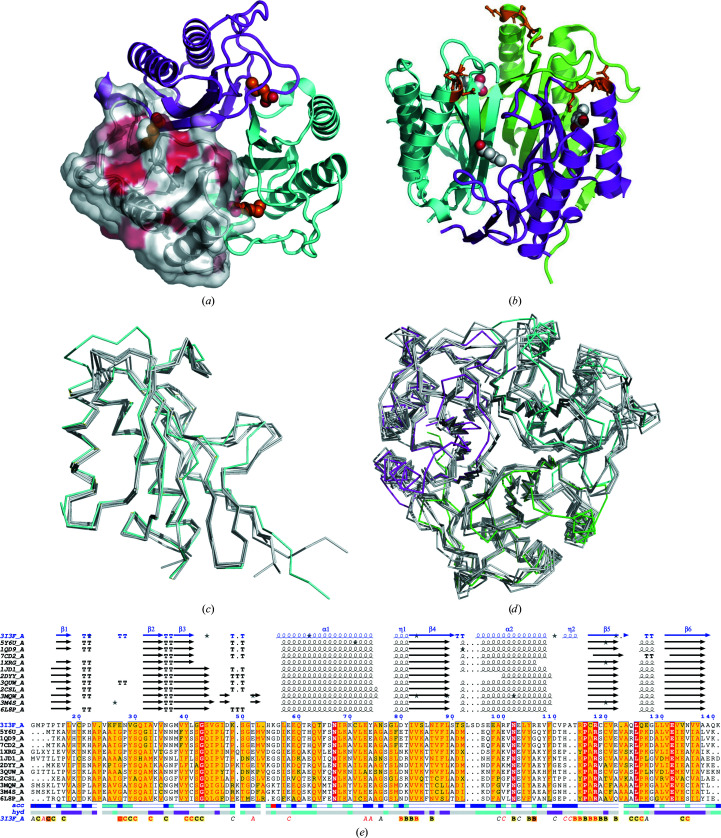
Structure of GilaA.00312.a (PDB entry 3i3f). (*a*) A GilaA.00312.a trimer with one monomer shown as a white surface; the identical residues in similar proteins are shown in red. (*b*) Alternative view of the trimer showing the unique insertion found in GilaA.00312.a as gold sticks. Endoribonuclease allosteric sites are identified by the modeled ligands in ball-and-stick representation. (*c*) Superposition of the GilaA.00312.a monomer (aquamarine) on the closest structures (gray). (*d*) A GilaA.00312.a trimer (aquamarine) superposed on the closest structures (gray). (*e*) *ENDScript* alignment of the closest structures. The secondary-structure elements shown are α-helices (α), 3_10_-helices (η), β-strands (β) and β-turns (TT). Identical residues are shown in red and similar residues in yellow. The same structures are used in (*c*), (*d*) and (*e*).

Each monomer of the hypothetical *G. lamblia* protein (GilaA.00312.a; PDB entry 3i3f) folds as a two-layer αβ sandwich. The quaternary structure is a homotrimer stabilized by seven hydrogen bonds and 99 nonbonded contacts per monomer. The trimer forms a β-barrel with core β-sheets surrounded by α-helices (Fig. 2 ▸ *a*). The largest interface between the monomers contains electron density for small molecules, which we built as two molecules of pentanoic acid and one of butanoic acid. The ligands have sufficient electron density, as indicated by composite omit maps (Supplementary Fig. S1), and their *B* factors are consistent with the contacting protein atoms. It is plausible that these ligands have dual conformations or could have been built with other small molecules (Supplementary Figs. S1 and S2). Nonetheless, the significance of these molecules is that they sit in the largest clefts in the structure. These largest clefts have volumes of ∼1850 Å^3^ and are consistent with the allosteric sites observed in other endoribonucleases.

*ENDScript* (Gouet *et al.*, 2003 ▸; Robert & Gouet, 2014 ▸) analysis was used to identify the most similar structures to GilaA.00312.a (Fig. 2 ▸). The most similar structures identified from the analysis were those of YabJ from *Bacillus subtilis* (PDB entries 5y6u, 1qd9 and 7cd2; Sinha *et al.*, 1999 ▸; Fujimoto *et al.*, 2021 ▸). A conserved hypothetical protein from *Clostridium thermocellum* Cth-2968 (PDB entry 1xrg) was identified as the next closest structure. Other similar structures include *Saccharomyces cerevisiae* homologous mitochondrial matrix factor 1 (PDB entry 1jd1; Deaconescu *et al.*, 2002 ▸), a putative translation-initiation inhibitor PH0854 from *Pyrococcus horikoshii* (PDB entry 2dyy), a putative endonuclease from *Entamoeba histolyca* (PDB entry 3mqw), *Saccharomyces cerevisiae* mitochondrial matrix protein Mmf1 (PDB entry 3quw; Pu *et al.*, 2011 ▸), TTHA0137 from *Thermus thermophilus* HB8 (PDB entry 2csl) and RidA from the Antarctic bacterium *Psychrobacter* sp. (PDB entry 6l8p; Kwon *et al.*, 2020 ▸). The similar structures identified by *ENDScript* analysis belong to the YjgF/YER057c/UK114 family of endoribonucleases with L-PSP topology (Kim *et al.*, 2018 ▸; Zhang *et al.*, 2010 ▸; Volz, 1999 ▸). Structural analysis with *PDBeFold* (http://www.ebi.ac.uk/msd-srv/ssm/; Krissinel & Henrick, 2004 ▸) and the *DALI* server (http://ekhidna2.biocenter.helsinki.fi/dali/; Holm, 2020 ▸) confirms that GilaA.00312.a has the structural features of the YjgF/YER057c/UK114 family of endoribo­nucleases (Figs. 2 ▸ and 3 ▸). Detailed results of the *DALI* and *PDBeFold* analysis are included in the supporting information.

**Figure 3:**
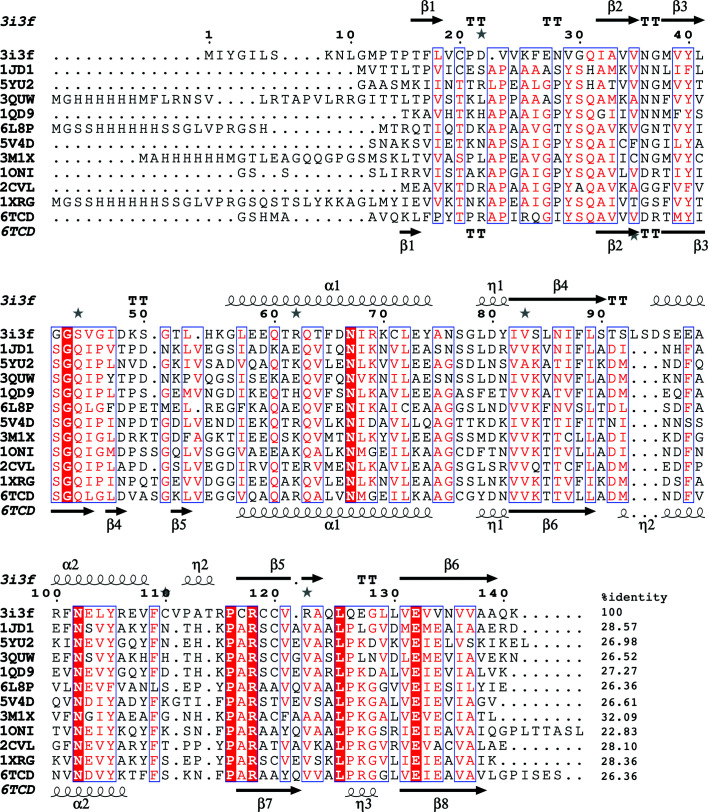
Structural and primary-sequence alignment of GilaA.00312.a and structurally similar YjgF/YER057c/UK114 endoribonucleases. The secondary-structure elements are shown as follows: α-helices are shown as large coils, 3_10_-helices ae shown as small coils labeled η, β-strands are shown as arrows labeled β and β-turns are labeled TT. Identical residues are shown on a red background; conserved residues are shown in red and conserved regions in blue boxes. This figure was generated using *ESPript* (Gouet *et al.*, 1999 ▸, 2003 ▸).

The YjgF/YER057c/UK114 family of endoribonucleases belong to the superfamily of proteins known as endoribo­nuclease L-PSP/chorismate mutase-like (IPR013813). These are homotrimeric proteins in which the intermolecular cavity forms putative allosteric binding sites for small molecules (Mistiniene *et al.*, 2003 ▸; Burman *et al.*, 2003 ▸, 2007 ▸; Miyakawa *et al.*, 2006 ▸). The superfamily includes two broadly defined families: YjgF/YER057c/UK114 and AroH chorismate mutases. The YjgF/YER057c/UK114 family are found in bacteria, archaea and eukaryotes (Lambrecht *et al.*, 2012 ▸), while AroH chorismate mutases are only found in bacteria. Specific members of the superfamily are YjgF (which was renamed RidA), which is known to deaminate reactive enamine/imine intermediates in pyridoxal 5′-phosphate (PLP)-dependent enzyme reactions (Lambrecht *et al.*, 2012 ▸), and the yeast growth inhibitor YER057c, which has roles in the regulation of metabolic pathways and cell differentiation (Kim *et al.*, 2001 ▸). Other members include UK114/L-PSP (liver perchloric acid-soluble protein), mammalian translational inhibitor proteins and endoribonucleases that directly affect mRNA translation by inducing disaggregation of the reticulo­cyte polysomes into 80S ribosomes (Morishita *et al.*, 1999 ▸). RutC is essential for the ability of *E. coli* to use uracil as a sole nitrogen source and possibly by reducing aminoacrylate peracid to aminoacrylate (Kim *et al.*, 2010 ▸), while *B. subtilis* YabJ is required for adenine-mediated repression of purine-biosynthetic genes (Sinha *et al.*, 1999 ▸). The structural neighbors of GilaA.00312.a are all members of the YjgF/YER057c/UK114 family and the overall structural topology is well conserved (Figs. 2 ▸ and 3 ▸). These closest structures share less than 32.1% sequence with GilaA.00312.a (Fig. 3 ▸).

GilaA.00312.a has a unique insertion of three residues (LSD) that differentiates it from its structural neighbors (Fig. 2 ▸). These residues follow Ser92 in the loop preceding α-helix 2 and form part of the access to the allosteric binding cavity of GilaA.00312.a (Figs. 2 ▸ and 3 ▸). Furthermore, the allosteric site of GilaA.00312.a differs from the conserved topology of its structural neighbors (Figs. 2 ▸ and 3 ▸). The significance of this observation will be investigated further since experimental evidence indicate that the metabolic functions of endoribo­nucleases are mediated by the allosteric site (Niehaus *et al.*, 2015 ▸).

## 4. Closing remarks

The structure of a hypothetical protein from *G. lamblia* (GilaA.00312.a) suggests that it belongs to the YjgF/YER057c/UK114 family, forming a trimer with allosteric active sites. Future studies are required to determine the ligands that bind to GilaA.00312.a and the specific mechanisms of its functions in the light of the observed unique structural features in its allosteric binding site.

## Supplementary Material

PDB reference: hypothetical protein from *Giardia lamblia*, 3i3f

Supplementary data including Supplementary Figures,. DOI: 10.1107/S2053230X21013595/ft5116sup1.pdf

## Figures and Tables

**Table 1:** Macromolecule-production information

Source organism	*Giardia lamblia* GL50803_14299
DNA source	Genomic DNA from Dr Ethan A. Merritt, University of Washington
Forward primer	GGGTCCTGGTTCGATGTTGACGGACTATCGCATCCG
Reverse primer	CTTGTTCGTGCTGTTTATTATACGAGGATGGTCCAGCAATCG
Cloning vector	pAVA0421
Expression vector	pAVA0421
Expression host	*E. coli* BL21(DE3)R3 Rosetta
Complete amino-acid sequence of the construct produced	MAHHHHHHMGTLEAQTQGPGSMIYGILSKNLGMPTPTFLVCPDVVKFENVGQIAVVNGMVYLGGSVGIDKSGTLHKGLEEQTRQTFDNIRKCLEYANSGLDYIVSLNIFLSTSLSDSEEARFNELYREVFCVPATRPCRCCVRAQLQEGLLVEVVNVVAAQK
Amino-acid sequence after 3C protease cleavage	GPGSMIYGILSKNLGMPTPTFLVCPDVVKFENVGQIAVVNGMVYLGGSVGIDKSGTLHKGLEEQTRQTFDNIRKCLEYANSGLDYIVSLNIFLSTSLSDSEEARFNELYREVFCVPATRPCRCCVRAQLQEGLLVEVVNVVAAQK

**Table 2:** Crystallization

Method	Sitting-drop vapor diffusion
Plate type	96-well Compact 300, Rigaku
Temperature (K)	290
Protein concentration (mg ml^−1^)	13.46
Buffer composition of protein solution	20 m*M* HEPES pH 7.0, 300 m*M* NaCl, 5% glycerol, 1 m*M* TCEP
Composition of reservoir solution	100 m*M* Tris pH 5.5, 25%(*w*/*v*) PEG 3350, 200 m*M* ammonium acetate
Volume and ratio of drop	0.4 µl protein solution plus 0.4 µl reservoir solution
Volume of reservoir (µl)	80

**Table 3:** Data collection and processing Values in parentheses are for the outer shell.

Diffraction source	SSRL beamline BL12-2
Wavelength (Å)	0.9795
Temperature (K)	100
Detector	ADSC Quantum 315R CCD
Space group	*I*4_1_22
*a*, *b*, *c* (Å)	119.90, 119.90, 104.59
α, β, γ (°)	90, 90, 90
Resolution range (Å)	39.41–1.35 (1.42–1.35)
No. of unique reflections	82643 (5692)
Completeness (%)	99.50 (96.60)
Multiplicity	6.30 (4.80)
〈*I*/σ(*I*)〉	13.50
*R*_r.i.m._ †	0.081 (5.40)
Overall *B* factor from Wilson plot (Å^2^)	14.0

† Estimated *R*_r.i.m._ = *R*_merge_[*N*/(*N* − 1)]^1/2^, where *N* is the data multiplicity.

**Table 4:** Structure solution and refinement Values in parentheses are for the outer shell.

Resolution range (Å)	37.35–1.35 (1.38–1.35)
Completeness (%)	99.6
No. of reflections, working set	82626 (5432)
No. of reflections, test set	4134 (260)
Final *R*_cryst_	0.176 (0.255)
Final *R*_free_	0.191 (0.264)
Cruickshank DPI	0.056
No. of non-H atoms	
Protein	2971
Ions	0
Ligand	20
Water	443
Total	3434
R.m.s. deviations	
Bonds (Å)	0.004
Angles (°)	0.893
Average *B* factors (Å^2^)	
Protein	13.5
Ligands	18.5
Water	23.7
Ramachandran plot	
Most favored (%)	97.81
Allowed (%)	2.19
Outlier (%)	0
