# Crystal structure of a hypothetical protein from *Giardia lamblia*. Corrigendum

**DOI:** 10.1107/S2053230X22001704

**Published:** 2022-02-28

**Authors:** Dylan K. Beard, Seonna Bristol, Kayla Cosby, Amber Davis, Courtney Manning, Lionel Perry, Lauren Snapp, Arian Toy, Kayla Wheeler, Jeremy Young, Bart Staker, Tracy L. Arakaki, Jan Abendroth, Sandhya Subramanian, Thomas E. Edwards, Peter J. Myler, Oluwatoyin A. Asojo

**Affiliations:** a Department of Chemistry and Biochemistry, Hampton University, 100 William R. Harvey Way, Hampton, VA 23668, USA; b Seattle Structural Genomics Center for Infectious Disease (SSGCID), Seattle, Washington, USA; c Center for Infectious Disease Research, formerly Seattle Biomedical Research Institute, 307 Westlake Avenue North Suite 500, Seattle, WA 98109, USA; d Labcorp Drug Development Inc., Princeton, NJ 08540, USA

**Keywords:** giardiasis, infectious diseases, travelers’ diarrhea, undergraduate education and training, corrigendum

## Abstract

The name of one of the authors in Beard *et al.* [(2022), *Acta Cryst.* F**78**, 59–65] is corrected.

## Abstract 2

The article by Beard *et al.* [(2022), *Acta Cryst.* F**78**, 59–65] is corrected.

In the article by Beard *et al.* (2022 ▸), the name of one of the authors was given incorrectly. The correct spelling is Sandhya Subramanian.
