# Supplementary material for: Crystal structure of a hypothetical protein from Giardia lamblia
Source: Acta Crystallogr F Struct Biol Commun. 2022 Jan 28;78(Pt 2):59–65. doi: 10.1107/S2053230X21013595 (PMC8805217; doi:10.1107/S2053230X21013595)
Supplement: Supplementary file 1 [file f-78-00059-sup1.pdf]

Acta Cryst F STRUCTURAL BIOLOGY COMMUNICATIONS

Volume 78 (2022)

Supporting information for article:

Crystal structure of a hypothetical protein from *Giardia lamblia*

Dylan Beard, Seonna Bristol, Kayla Cosby, Amber Davis, Courtney Manning, Lionel Perry, Lauren Snapp, Arian Toy, Kayla Wheeler, Jeremy Young, Bart L. Staker, David Dranow, Jan Abendroth, Sandhya Subrahamanian, Thomas E. Edwards, Peter J. Myler and Oluwatoyin A. Asojo

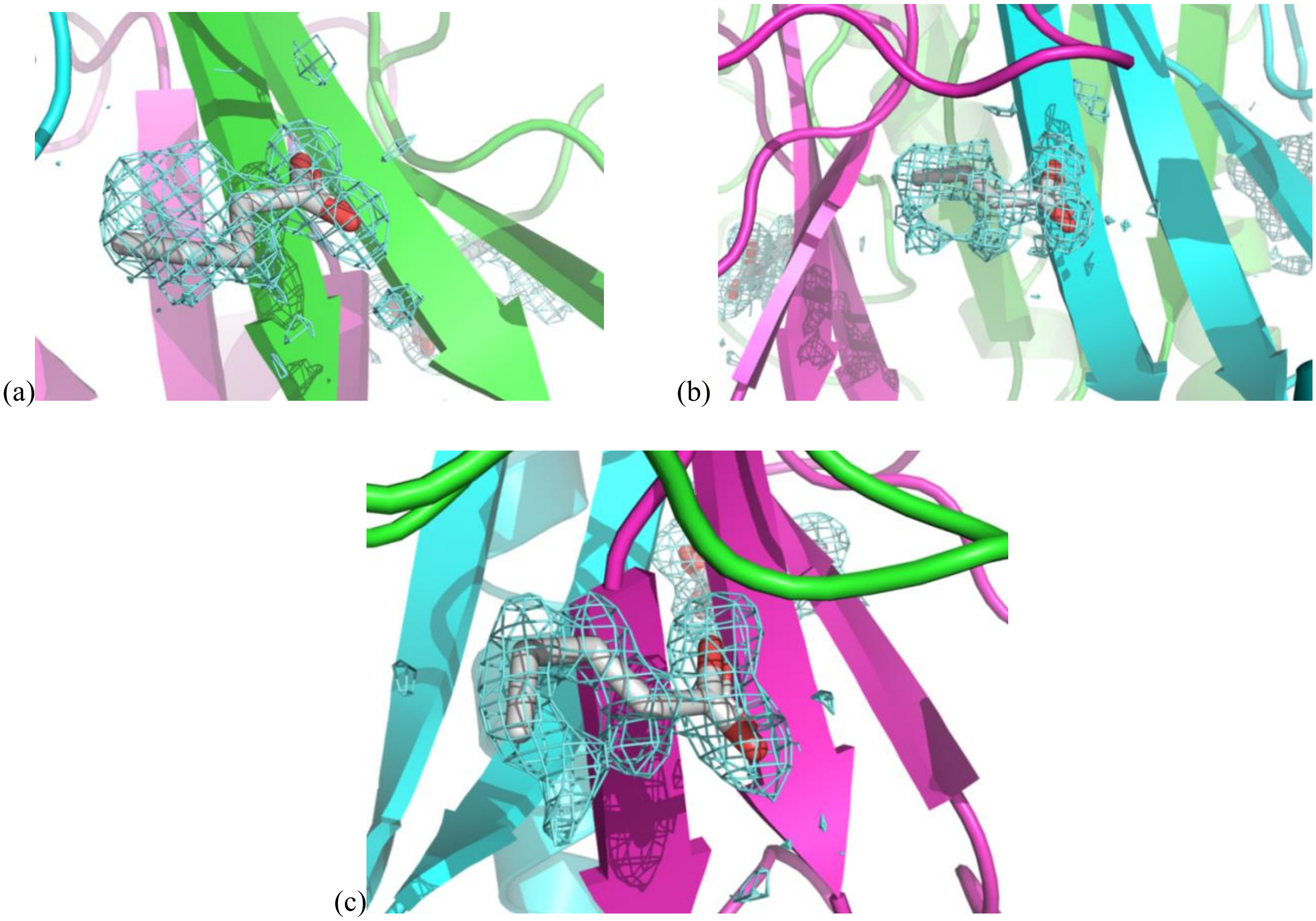

Figure S1 Composite omit maps of the ligands built into each of the three allosteric sites (a, b, c) contoured at 3.0σ, generated with CCP4 and image generated with PyMOL.

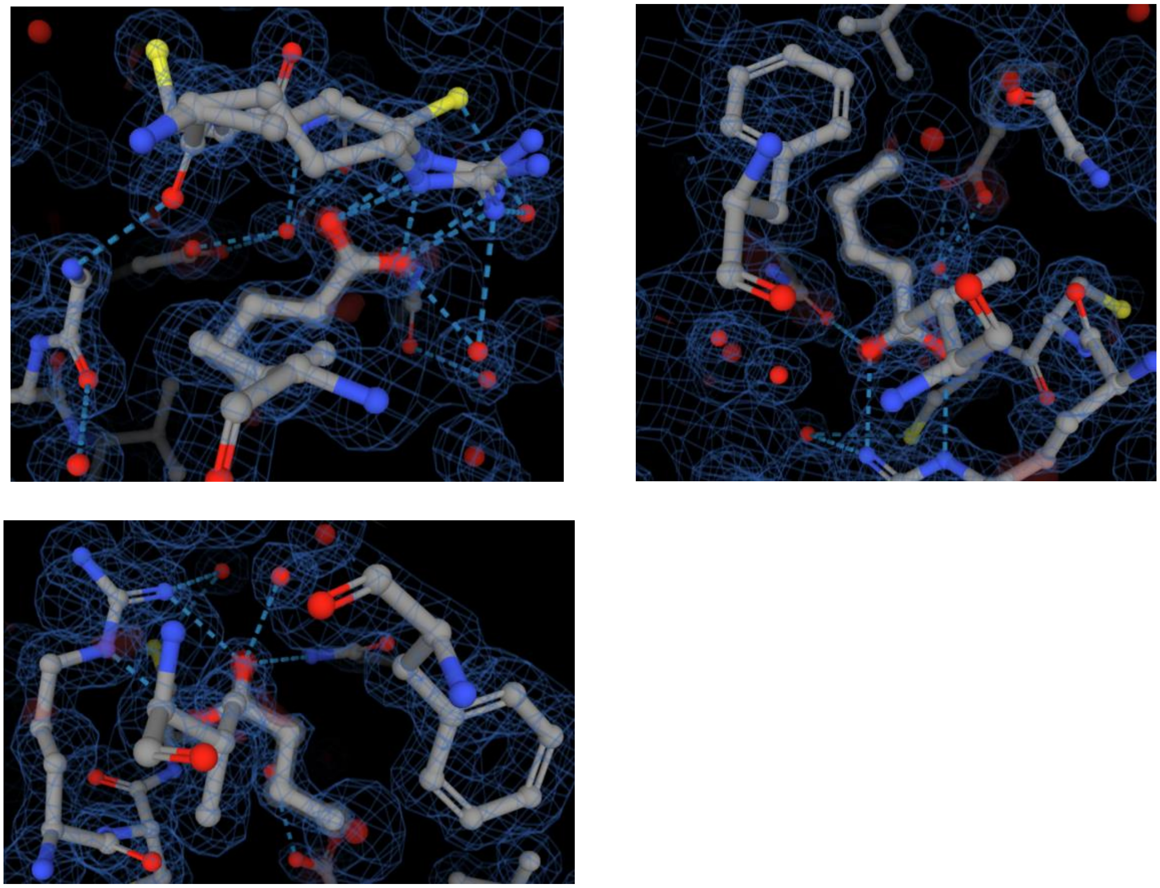

Figure S2 Representative quality of 2Fo-Fc electron density maps in the proximity to the three allosteric sites contoured at 1.2σ generated with Mol-*, the 3-D visualization tools of PDBe. (https://www.ebi.ac.uk/pdbe/about/news/introducing-mol-fast-interactive-3d-visualisation-your-browser-rcsb-pdb-and-pdbe).

Dali alignment: 3i3f

Each neighbour is shown in the pairwise Dali-alignment to 3i3fA. Inserted segments relative to the top structure are hidden. You can check the 'Expand gaps' option in the summary page to see the complete sequence of the matched proteins. Uppercase means structurally equivalent positions with 3i3fA. Lowercase means insertions relative to 3i3fA. The first part shows the amino acid sequences of the selected neighbours. The second part shows the secondary structure assignments by DSSP (H/h: helix, E/e: strand, L/l: coil). The most frequent amino acid type is coloured in each column.

Show Stacked Sequence Logos

```
                    :         :         :         :         |         :         :         :         :        100         :         :         :
0001 3i3fA   GMPTPTFLVCPDVVKFENVGQIAVVNGMVYLGGSVGIDKSGTLHKGLEEQTRQTFDNIRKCLEYANSGLDYIVSLNIFLSTSLSDSEEARFNELYREVFCVPATRPCRCCVRAQLQEGLLVEVVNVVAAQK
0002 3i3fA   GMPTPTFLVCPDVVKFENVGQIAVVNGMVYLGGSVGIDKSGTLHKGLEEQTRQTFDNIRKCLEYANSGLDYIVSLNIFLSTSLSDSEEARFNELYREVFCVPATRPCRCCVRAQLQEGLLVEVVNVVAAQK
0003 1jd1C   --TTLTPVICESAPAAASYSHAMKVNNLIFLSGQIPVTPDNKLVESIADKAEQVIQNIKNVLEASNSSLDRVVKVNIFLAD---INHFAEFNSVYAKYFNT--HKPARSCVAVALPLGVDMEME-AIAAER
0004 5yu2E   ----MKIINTTRLPEAGPYSHATVVNGMVYTSGQIPLNVDGKIVSDVQAQTKQVLENLKVVLEEAGSDLNSVAKATIFIK--DMND-FQKINEVYGQYFNE--HKPARSCVEVRLPKDVKVEIE-LVSKIK
0005 3quwA   ---TLTPVSTKLAPPAASYSQAMKANNFVYVSGQIPYTPDNKPVQSISEKAEQVFQNVKNILAESNSSLDNIVKVNVFLAD----KNFAEFNSVYAKHFHT--HKPARSCVGVSLPLNVDLEME-VIAVEK
0006 1qd9B   ----TKAVHTKHAPAAIGYSQGIIVNNMFYSSGQIPLTPSGEMVGDIKEQTHQVFSNLKAVLEEAGASFETVVKATVFIA--DMEQ-FAEVNEVYGQYFDT--HKPARSCVEVRLPKDALVEIE-VIALVK
0007 5yu2B   ----MKIINTTRLPEALPYSHATVVNGMVYTSGQIPLNVDGKIVSDVQAQTKQVLENLKVVLEEAGSDLNSVAKATIFIKD---MNDFQKINEVYGQYFNE--HKPARSCVEVRLPKDVKVEIE-LVSKIK
0008 6l8pB   ----RQTIQTDKAPAAGTYSQAVKVGNTVYISGQLGFDPTMELREGFKAQAEQVFENIKAICEAAGGSLNDVVKFNVSLTD---LSDFAVLNEVFVANLSE--PYPARAAVQVALPKGGVVEIE-SILYI-
0009 3m1xA   --SKLTVVASPLAPEAVGYSQAIICNGXVYCSGQIGLDRKGDFAKTIEEQSKQVMTNLKYVLEEAGSSMDKVVKTTCLLAD----KDFGVFNGIYAEAFGN--HKPARACFAALPKGALVEVE-CIATL-
0010 6tcdE   AAVQKLFPYTPRAPIRQIYSQAVVVDRTMYISGQLGLDVAGKLVEGVQAQARQALVNMGEILKAAGCGYDNVVKTTVLLADMN---DFVNVNDVYKTFFSK--NFPARAAYQVALPRGGLVEIE-AVAVLG
0011 5v4dA   --NAKSVIETKNAPSAGPYSQAICFNGILYASGQIPINPTGDLVEDIEKQTRQVLKNIDAVLLQAGTTKDKIVKTTIFITN----NNSSQVNDIYADYFKGT-IFPARSTVEVALPKGALVEIE-VIAGV-
0012 1oniF   SSLIRRVISTAKAPGAGPYSQAVLVDRTIYISGQIGMDPSGQLVSGVAEEAKQALKNMGEILKAAGCDFTNVVKTTVLLAD----NDFNTVNEIYKQYFKS--NFPARAAYQVALPKGSRIEIE-AVAIQG
0013 1xrgA   ---YIEVVKTNKAPEAIGYSQAIVTGSFVYTSGQIPINPQGEVVGGIEEQAKQVLENLKNVLEAAGSSLNKVVKTTVFIK--DXDS-FAKVNEVYAKYFSE--PYPARSCVEVKLPKGVLIEIE-AVAIK-
0014 1oniH   SSLIRRVISTAKAPGAIGYSQAVLVDRTIYISGQIGMDPSGQLVSGVAEEAKQALKNMGEILKAAGCDFTNVVKTTVLLAD----NDFNTVNEIYKQYFKS--NFPARAAYQVALPKGSRIEIE-AVAIQG
0015 6tccA   SSIIRKIINTSKAPAAGPYSQAVVVDRTMYVSGQLGMDPAGQLVEGVQAQTKQALVNMGEILKEAGCGYDSVVKTTVLLAD----NDFASVNDVYKTFFSS--SFPARAAYQVALPRGGLVEIE-AVAVLG
0016 1qahA   SSIIRKVISTSKAPAAGAYSQAVLVDRTIYVSGQIGMDPSGQLVPGVAEEAKQALKNLGEILKAAGCDFTNVVKTTVLLAD----NDFGTVNEIYKTYFQG--NLPARAAYQVALPKGSRIEIE-AIAVQG
0017 1oniE   SSLIRRVISTAKAPGAGPYSQAVLVDRTIYISGQIGMDPSGQLVSGVAEEAKQALKNMGEILKAAGCDFTNVVKTTVLLAD----NDFNTVNEIYKQYFKS--NFPARAAYQVALPKGSRIEIE-AVAIQG
0018 3l7qD   -----KKIHTDKAPAAIGYVQGKIVGNLLFASGQVPLSPEGQVITTIEEQTQQVLKNISAILTEAGTDFDHVVKTTCFLSD----DDFVPFNEVYATAFKS--DFPARSAVEVRLPKDVKIEIE-VIAELI
0019 2dyyG   ----KEVIFTENAPKPIGYSQAIKAGNFLFIAGQIPIDPKGEIVGDIKDQTRQVLENIKAILEEAAGYSLNDVIKVTVYLKD---MNDFAKMNEVYAEYFGE--SKPARVAVEVRLPKDVLIEIE-AIAYKE
0020 1nq3F   -SLVRRIISTAKAPAAIGYSQAVLVDRTIYISGQLGMDPSGQLVPGVVEEAKQALTNIGEILKAAGCDFTNVVKATVLLAD----NDFSAVNDVYKQYFQS--SFPARAAYQVALPKGGRVEIE-AIAVQG
0021 2cslE   ----XEAVKTDRAPAAIGYAQAVKAGGFVFVSGQIPLAPDGSLVEGIRVQTERVXENLKAVLEAAGSGLSRVVQTTCFLAD---XEDFPGFNEVYARYFTP--PYPARATVAVALPRGVRVEVA-CVALAE
0022 3k0tC   ----KTVITSDKAPAAIGYSQAIKAGNTVYXSGQIPLDPSXELVEGIEAQITQVFENLKSVAQAAGGSFKDIVKLNIFL-TDLG--HFAKVNEIXGSYFSQ--PYPARAAIGVALPRGAQVEXD-AILVIE
0023 2uykA   ----KKIIETQRAPGAGPYVQGVDLGSMVFTSGQIPVXPTGEIPADVQDQARLSLENVKAIVVAAGLSVGDIIKMTVFITD---LNDFATINEVYKQFFDE--TYPTRSCVQVRLPKDVKLEIE-AIAVRS
0024 2uynA   ----KKIIETQRAPGAGPYVQGVDLGSMVFTSGQIPVXPTGEIPADVQDQARLSLENVXAIVVAAGLSVGDIIKMTVFITD---LNDFATINEVYKQFFDE--TYPTRSCVQVRLPKDVKLEIE-AIAVRS
0025 3r0pF   --TNKAIIHSDNAPAAIGYSQAVKVNNTVYLSGQIPLDPVMQLVGDFAVQAHQVFKNLRAVCEAAGGGLRDIVKLNVYLTD----ANFPIVNEVMGQYFQA--PYPARAAIGIQLPRASLIEAD-GIMVI-
0026 2uypC   ----KKIIETQRAPGAGPYVQGVDLGSMVFTSGQIPVXPQGEIPADVQDQARLSLENVKAIVVAAGLSVGDIIKMTVFITD---LNDFATINEVYKQFFDE--TYPTRSCVQVRLPKDVKLEIE-AIAVRS
0027 3vczA   ----TKVLHTDSAPAAIGYIQGVDLGNMVLTSGQIPVNPAGEVPADIAAQARQSLDNVKAVVEASGLTVGDIVKMTVFVKD----NDFGTVNEVYGNFFDNVAHYPARSCVEVRLPKDVGIEIE-AIAVR-
0028 2b33A   ---MKRFVETDKAPKAIGYSQAVVVGNMMFVSGQIPIDPTGELVQTIEEKTERVLENLKAILEAGGFSLKDVVKVTVFTTS---MDYFQRVNEVYSRYFGD--HRPARSFVAVQLPRNVEIEIE-AIAVKE
0029 3lmeL   ---SLKIIAPTKTITPGTWSIGARAGDFVFIGGMHGTDRVGKMVDGDEARIRRMFDNMLAAAEAAGATKADAVRLTVFVT--DVAKYRPVVNKVQKDIWGDG-PYPPRTVLQVALDQGDIAEID-GTFYAP
0030 3r0pB   ---NKAIIHSDNAPAAIGYSQAVKVNNTVYLSGQIPLDPVMQLVGDFAVQAHQVFKNLRAVCEAAGGGLRDIVKLNVYLTD----ANFPIVNEVMGQYFQA--PYPARAAIGIQLPRASLIEAD-GIMVI-
0031 3lmeE   ---SLKIIAPTKTITPSGWSIGARAGDFVFIGGMHGTDRVGKMVDGDEARIRRMFDNMLAAAEAAGATKADAVRLTVFVT--DVAKYRPVVNKVQKDIWGDG-PYPPRTVLQVALDQGDIAEID-GTFYAP
0032 6izhI   ----NSAKLVEGKAKPGSFPHVKRAGDFLFVSGTSSRRPDNTFVGNIELQTREVISNIRDILQSVGADLGDVVEVCSYLVN---MNDFAAYNKVYAEFFDA--TGPARTTVAVQLPHQLVIEIK-VVAYKP
0033 3kjkF   --XDIRYFG------TPRYSEAVGANGLIFLSGXVPE-----NGETAAEQTADVLAQIDRWLAECGSDKAHVLDAVIYLRDXG---DYAEXNGVWDAWVAA-GRTPARACVEARLAREWRVEIK-ITAVKR
0034 3kjjF   -HXDIRYFG------TPRYSEAVGANGLIFLSGXVPE-----NGETAAEQTADVLAQIDRWLAECGSDKAHVLDAVIYLRDXG---DYAEXNGVWDAWVAA-GRTPARACVEARLAREWRVEIK-ITAVKR
0035 2dyyC   ---MKEVIFTENAPKPIGYSQAIKAGNFLFIAGQIPIDPKGEIVGDIKDQTRQVLENIKAILEEAAGYSLNDVIKVTVYLKD----------NEVYAEYFGE---KPARVAVEVRLPKDVLIEIE-AIAYKE
0036 3k12D   ---HIERFE-------KRRAEXALHGNTVYIGGQVAD----DPSGDIQDQTRQILENIDRLLQSVGSDRGQVLSVRILLAHRE---DYAGLNQVWDQWFPE-GRAPTRACSLAELIDRWRVEXI-VVAARE
0037 7cd2L   -------------------SQGIIVNNMFYSSGQIPLTPSGEMVGDIKEQTHQVFSNLKAVLEEAGASFETVVKATVFIAD----EQFAEVNEVYGQYFDT--HKPAFCVEVARLPKDALVEIE-VIALVK
0038 7cd2H   --------------------QGIIVNNMFYSSGQIPLTPSGEMVGDIKEQTHQVFSNLKAVLEEAGASFETVVKATVFI-ADME--QFAEVNEVYGQYFDT--HKPAFCVEVARLPKDALVEIE-VIALVK
0039 2ig8C   -MTAVRRIRAAALPD-ASWSNALLVGEELVMSGMTA----HPATLDAHAQALVVLGKVKALLEAAGGHVGNLYKLNVYVTRIA---DKDAIGRARQEFFAGQGTFPASTLVEVGLVFELLVEID-AWARLD
0040 3gtzC   --LSIVRIDAEDRWS-----DVVIYNNTLWY-TGVPE----NLDADAFEQTANTLAQIDAVLEKQGSSKSRILDATIFLSDKA---DFAAMNKAWDAWVVA-GHAPVRCTVQAGLMPKYKVEIK-IVAAV-
0041 5v4fA   ------------------------SGRTLHIAGQVAWNANGELVGDLLAQTQQVLANLKEVLRYAGATPADVVRLRTYVVN-HSPANLAAICAQIGAFYEGA-DPAANSFIGVALALELLIEIE-ATACL-
0042 5a3kB   RSGGHVHIENPRQVPAYRFARATYLPGQVFVSGTASVLGHETAHEDLVKQCRLALENIELVISG-GHGLTALRNIKVYVRRSE---DVPAVREICREAF----PDADIVYLTVDVSSDLLVEIE-GVVM--
                    :         :         :         :         |         :         :         :         :        100         :         :         :
0001 3i3fA   LLLLLEEELLLLLLLLLLLLLLEEEELLEEEELLLLLLLLLLLLLLHHHHHHHHHHHHHHHHHHHHLLLLHHHEEEEEEEEEELLLLHHHHHHHHHHHHHHHLLLHHHLLEEEEEEELLLLLLLEEEEEEEEEELL
0002 3i3fA   LLLLLEEELLLLLLLLLLLLLLEEEELLEEEELLLLLLLLLLLLLLHHHHHHHHHHHHHHHHHHHHLLLLHHHEEEEEEEEEELLLLHHHHHHHHHHHHHHHLLLHHHLLEEEEEEELLLLLLLEEEEEEEEEELL
0003 1jd1C   --LLEEEELLLLLLLLLLLLLLEEEELLEEEEEEEELLLLLLLLLLLLHHHHHHHHHHHHHHHHHHHHLLLLHHHEEEEEEEEEELL---HHHHHHHHHHHHHHHLLL--LLLEEEEEEELLLHHHLLEEEE-EEEELL
0004 5yu2E   ----LEEELLLLLLLLLLLLLLEEEELLEEEELLLLLLLLLLLLLLLHHHHHHHHHHHHHHHHHHHHLLLLHHHEEEEEEEEEL--LHHH-HHHHHHHHHHHHHLLL--LLLEEEEEEELLLHHHLLEEEE-EEEELL
0005 3quwA   ---LEEEELLLLLLLLLLLLLLEEEELLEEEEEEEELLLLLLLLLLLLHHHHHHHHHHHHHHHHHHHHLLLLHHHEEEEEEEEEELL----HHHHHHHHHHHHHHLLL--LLLEEEEEEELLLHHHLLEEEE-EEEELL
0006 1qd9B   ----LEEELLLLLLLLLLLLLLEEEELLEEEELLLLLLLLLLLLLLLHHHHHHHHHHHHHHHHHHHHLLLLHHHEEEEEEEEEL--LHHH-HHHHHHHHHHHHHLLL--LLLEEEEEEELLLHHHLLEEEE-EEEELL
0007 5yu2B   ----LEEELLLLLLLLLLLLLLEEEELLEEEELLLLLLLLLLLLLLLHHHHHHHHHHHHHHHHHHHHLLLLHHHEEEEEEEEEELL---HHHHHHHHHHHHHHHLLL--LLLEEEEEEELLLHHHLLEEEE-EEEELL
0008 6l8pB   ----LEEELLLLLLLLLLLLLLEEEELLEEEEEEEELLEELLLEELLLHHHHHHHHHHHHHHHHHHHHLLLLHHHEEEEEEEEEELL---HHHHHHHHHHHHHHHLLL--LLLEEEEEEELLLHHHLLEEEE-EEEEL-
0009 3m1xA   --LLEEEELLLLLLLLLLLLLLEEEELLEEEEEEEEELEELLLEELLLHHHHHHHHHHHHHHHHHHHHLLLLHHHEEEEEEEEEELL----HHHHHHHHHHHHHHLL--LLLEEEEEEELLLHHHLLEEEE-EEEEL-
0010 6tcdE   LLLLEELLLLLLLLLLLLLLLLEEEELLEEEEEEEEELEELLLEELLLHHHHHHHHHHHHHHHHHHHHLLLLHHHEEEEEEEEEELLLL---LHHHHHHHHLLLLLL--LLLEEEEEEELLLHHHLLEEEE-EEEELL
0011 5v4dA   --LLLEEELLLLLLLLLLLLLLEEEELLEEEELLLLLLLLLLLLLLLHHHHHHHHHHHHHHHHHHHHLLLLHHHEEEEEEEEEELL----HHHHHHHHHHHHHHHLLL-LLLEEEEEEELLLHHHLLEEEE-EEEEL-
0012 1oniF   LLEEEEEELLLLLLLLLLLLLLEEEELLEEEEEEEELLLLLLLLLLLLHHHHHHHHHHHHHHHHHHHHLLLLHHHEEEEEEEEEELL----HHHHHHHHHHLLLLLL--LLLEEEEEEELLLHHHLLEEEE-EEEELL
0013 1xrgA   ---LLEEELLLLLLLLLLLLLLEEEELLEEEELLLLLLLLLLLLLLLHHHHHHHHHHHHHHHHHHHHLLLLHHHEEEEEEEEEL--LHHH-HHHHHHHHHLLLLL--LLLEEEEEEELLLHHHLLEEEE-EEEEL-
0014 1oniH   LLEEEEEELLLLLLLLLLLLLLEEEELLEEEEEEEELLLLLLLLLLLLHHHHHHHHHHHHHHHHHHHHLLLLHHHEEEEEEEEEELL----HHHHHHHHHHLLLLLL--LLLEEEEEEELLLHHHLLEEEE-EEEELL
0015 6tccA   LLLLEEEELLLLLLLLLLLLLLEEEELLEEEEEEEEELLLLLLLLLLLHHHHHHHHHHHHHHHHHHHHLLLLHHHEEEEEEEEEELL----HHHHHHHHHHHHHHHLLL--LLLEEEEEEELLLHHHLLEEEE-EEEELL
0016 1qahA   LLLLEEEELLLLLLLLLLLLLLEEEELLEEEEEEEELLLLLLLLLLLLHHHHHHHHHHHHHHHHHHHHLLLLHHHEEEEEEEEEELL----HHHHHHHHHHHLLLLL--LLLEEEEEEELLLHHHLLEEEE-EEEELL
0017 1oniE   LLEEEEEELLLLLLLLLLLLLLEEEELLEEEEEEEELLLLLLLLLLLHHHHHHHHHHHHHHHHHHHHHLLLLHHHEEEEEEEEEELL----HHHHHHHHHHLLLLLL--LLLEEEEEEELLLHHHLLEEEE-EEEELL
0018 3l7qD   -----LEELLLLLLLLLLLLLLEEEELLEEEELLLLLLLLLLLLLLLHHHHHHHHHHHHHHHHHHHHLLLHHHEEEEEEEEEELL----HHHHHHHHHHHHHHHLLL--LLLEEEEEEELLLHHHLLEEEE-EEEELL
0019 2dyyG   ----LEEELLLLLLLLLLLLLLEEEELLEEEEEEEELLLLLLLLLLLLHHHHHHHHHHHHHHHHHHHHLLLLHHHEEEEEEEEEELL---LLLHHHHHHHHHHHHHLL--LLLEEEEEEELLLHHHLLEEEE-EEEELL
0020 1nq3F   -LEEEEEELLLLLLLLLLLLLLEEEELLEEEEEEEEELLLLLLLLLLLHHHHHHHHHHHHHHHHHHHHLLLLHHHEEEEEEEEEELL----HHHHHHHHHHLLLLLL--LLLEEEEEEELLLHHHLLEEEE-EEEELL
0021 2cslE   ----LEELLLLLLLLLLLLLLLEEEELLEEEEEEEELLLLLLLLLLLLHHHHHHHHHHHHHHHHHHHHLLLLHHHEEEEEEEEEELL---HHHHHHHHHHHHHHHLLL--LLLEEEEEEELLLHHHLLEEEE-EEEELL
0022 3k0tC   ----LEEELLLLLLLLLLLLLLEEEELLEEEEEEEELLEELLLEELLLHHHHHHHHHHHHHHHHHHHHLLLLHHHEEEEEEEEEE-LLHH--HHHHHHHHHHHHHHLLL--LLLEEEEEEELLLHHHLLEEEE-EEEELL
0023 2uykA   ----LEEELLLLLLLLLLLLLLEEELLLEEEELLLLLLLLLLLLLLLHHHHHHHHHHHHHHHHHHHHLLLLHHHEEEEEEEEEELL---HHHHHHHHHHHHHHHHHH--LLLEEEEEEELLLHHHLLEEEE-EEEELL
0024 2uynA   ----LEEELLLLLLLLLLLLLLEEELLLEEEELLLLLLLLLLLLLLLHHHHHHHHHHHHHHHHHHHHLLLLHHHEEEEEEEEEELL---HHHHHHHHHHHHHHHHHH--LLLEEEEEEELLLHHHLLEEEE-EEEELL
0025 3r0pF   --LLEEEELLLLLLLLLLLLLLEEEELLEEEEEEEELLLLLLLLLLLLHHHHHHHHHHHHHHHHHHHHLLLLHHHEEEEEEEEEELL----HHHHHHHHHHLLLLLL--LLLEEEEEEELLLHHHLLEEEE-EEEEL-
0026 2uypC   ----LEEELLLLLLLLLLLLLLEEELLLEEEELLLLLLLLLLLLLLLHHHHHHHHHHHHHHHHHHHHLLLLHHHEEEEEEEEEELL---HHHHHHHHHHHHHHHHHH--LLLEEEEEEELLLHHHLLEEEE-EEEELL
0027 3vczA   ----LEEELLLLLLLLLLLLLLEEELLLEEEELLLLLLLLLLLLLLLHHHHHHHHHHHHHHHHHHLLLLLHHHEEEEEEEEEELL----HHHHHHHHHHHHHHHHLLLLLLEEEEEEELLLHHHLLEEEE-EEEEL-
0028 2b33A   ---LLEEELLLLLLLLLLLLLLEEEELLEEEEEEEELLLLLLLLLLLLHHHHHHHHHHHHHHHHHHHHLLLLHHHEEEEEEEEEELL---HHHHHHHHHHHHHHHHLL--LLLEEEEEEELLLHHHLLEEEE-EEEELL
0029 3lmeL   ---LLEELLLLLLLLLLLLLLLEEEELLEEEEEEEELLLLLLLLLLLLHHHHHHHHHHHHHHHHHHLLLLLHHHEEEEEEEEEEL--LHHHHHHHHHHHHHHHHLLL-LLLLEEEEELLLHHHLLEEEE-EEEELL
0030 3r0pB   ---LEEEELLLLLLLLLLLLLLEEEELLEEEEEEEELLLLLLLLLLLLHHHHHHHHHHHHHHHHHHHHLLLLHHHEEEEEEEEEELL----HHHHHHHHHHHHHHHLLL--LLLEEEEEEELLLHHHLLEEEE-EEEEL-
0031 3lmeE   ---LLEELLLLLLLLLLLLLLLEEEELLEEEEEEEELLLLLLLLLLLLHHHHHHHHHHHHHHHHHHHHLLLLHHHEEEEEEEEEEL--LHHHHHHHHHHHHHHHHLLL-LLLLEEEEELLLHHHLLEEEE-EEEELL
0032 6izhI   ----LLEELLLLLLLLLLLLLLEEEELLEEEEEEEELLLLLLLLLLLLHHHHHHHHHHHHHHHHHHHHLLLLHHHEEEEEEEEEELL---HHHHHHHHHHHHHHHLLL--LLLEEEEEEELLLLLLLEEEE-EEEELL
0033 3kjkF   --LLEEEEE------LLLLEEEEEELLEEEELLLLLL-----LLLLHHHHHHHHHHHHHHHHHHHHLLLLHHHEEEEEEEEEELLHH---HHHHHHHHHHHHHLLL-LLLLEEEEEEELLLLLLLEEEE-EEEELL
0034 3kjjF   -LLLEEEEE------LLLLEEEEEELLEEEELLLLLL-----LLLLHHHHHHHHHHHHHHHHHHHHLLLLHHHEEEEEEEEEELLHH---HHHHHHHHHHHHHLLL-LLLLEEEEEEELLLLLLLEEEE-EEEELL
0035 2dyyC   ---LEEEELLLLLLLLLLLLLLEEEELLEEEEEEEELLLLLLLLLLLLHHHHHHHHHHHHHHHHHHHHLLLLHHHEEEEEEEEEELL----------LHHHHHHHLL---LLEEEEEEELLLHHHLLEEEE-EEEELL
0036 3k12D   ---LLEEEE-------LLLEEEEEELLEEEEEEEELLL----LLLLLHHHHHHHHHHHHHHHHHHHHLLLLLLLEEEEEEEEEELLHH---HHHHHHHHHHHHHLLL-LLLLEEEEEEELLLLLLLEEEE-EEEELL
0037 7cd2L   -------------------LLEEEELLEEEELLLLLLLLLLLLLLLHHHHHHHHHHHHHHHHHHHHLLLHHHEEEEEEEEEELL----HHHHHHHHHHHHHHLLL--LLLLEEEEEELLLLLLLEEEE-EEEELL
0038 7cd2H   --------------------LLEEELLEEELLLLLLLLLLLLLLLLHHHHHHHHHHHHHHHHHHLLLLHHHEEEEEEEEEE-LLHH--HHHHHHHHHHHHHLLL--LLLLEEEEEELLLLLLLEEEE-EEEELL
0039 2ig8C   -LLLEEELLLLLLLL-LLLLLEEEELLEEEELLLLL----LLHHLLHHHHHHHHHHHHHHHHHHHHLLLLHHHEEEEEEEEEELLHH---HHHHHHHHHHHHLLLLLLLLEEEEEEELLLLLLLEEEE-EEEELL
0040 3gtzC   --LLLEEELLLLLLL-----LEEEELLEEEE-EELLL----LLLLLHHHHHHHHHHHHHHHHHHHHHLLLLHHHEEEEEEEEEELLHH---HHHHHHHHHHHHHLLL-LLLLEEEEEEELLLLLLLEEEE-EEEEL-
0041 5v4fA   ------------------------LLLEEEEEEEELLLLLLLLLLLHHHHHHHHHHHHHHHHHHHHLLLHHHEEEEEEEEEEL-LLHHHHHHHHHHHHHHLLLL-LLLLLEEEELLLLLLLEEEE-EEEEL-
0042 5a3kB   LLLLLEEELLLLLLLHHLLLLEEEELLEEEEEEEELLEELLEELLLLHHHHHHHHHHHHHHHHLLH-LLLHHHEEEEEEEEEELLLL---LHHHHHHHHHLLL----LLLLEEEEELLLLLLLLEEEE-EEEL--
```

PDBe Fold v2.59. (src3) 14 Apr 2014 result file.

RESULT SUMMARY

##	Q-score	P-score	Z-score	RMSD	Nalgn	Nsse	Ngaps	Seq-%	Nmd	Nres-Q	Nsse-Q	Nres-T	Nsse-T	Query	Target
1	1	37.19	18.28	0.000	131	9	0	1	0	131	9	131	9	PDB 3i3f:A	PDB 3i3f:A
2	0.9597	25.28	15.01	0.404	128	9	0	1	0	131	9	128	9	PDB 3i3f:A	PDB 3i3f:C
3	0.9583	25.43	15.05	0.420	128	9	0	1	0	131	9	128	9	PDB 3i3f:A	PDB 3i3f:B
4	0.7262	12.69	10.65	1.274	118	8	4	0.2797	0	131	9	124	9	PDB 3i3f:A	PDB 5yu2:B
5	0.7243	9.938	9.428	1.321	119	8	4	0.2773	0	131	9	125	9	PDB 3i3f:A	PDB 3quw:A
6	0.7227	12.58	10.6	1.294	118	8	4	0.2797	0	131	9	124	9	PDB 3i3f:A	PDB 5yu2:E
7	0.7191	12.58	10.6	1.315	118	8	4	0.2797	0	131	9	124	9	PDB 3i3f:A	PDB 5yu2:D
8	0.7183	10.61	9.738	1.421	120	8	4	0.2417	0	131	9	125	9	PDB 3i3f:A	PDB 2dyy:G
9	0.7178	12.96	10.76	1.322	118	8	4	0.2797	0	131	9	124	9	PDB 3i3f:A	PDB 5yu2:F
10	0.717	12.62	10.62	1.326	118	8	4	0.2797	0	131	9	124	9	PDB 3i3f:A	PDB 5y6u:B
11	0.7132	12.54	10.59	1.348	118	8	4	0.2797	0	131	9	124	9	PDB 3i3f:A	PDB 5y6u:C
12	0.7095	12.62	10.62	1.369	118	8	4	0.2797	0	131	9	124	9	PDB 3i3f:A	PDB 5y6u:A
13	0.7087	12.77	10.68	1.380	120	8	4	0.275	0	131	9	128	9	PDB 3i3f:A	PDB 5yu2:A
14	0.7081	10.78	9.816	1.341	117	8	4	0.265	0	131	9	123	9	PDB 3i3f:A	PDB 6l8p:B
15	0.7071	9.576	9.259	1.314	117	8	4	0.265	0	131	9	124	9	PDB 3i3f:A	PDB 6l8p:A
16	0.7062	12.65	10.64	1.323	118	8	4	0.2797	0	131	9	126	9	PDB 3i3f:A	PDB 5yu2:C
17	0.706	9.356	9.151	1.361	119	7	5	0.3193	0	131	9	127	9	PDB 3i3f:A	PDB 3m1x:A
18	0.7025	9.67	9.474	1.340	117	7	5	0.2479	0	131	9	124	8	PDB 3i3f:A	PDB 3l7q:B
19	0.7012	9.273	9.285	1.348	117	7	5	0.2479	0	131	9	124	8	PDB 3i3f:A	PDB 3l7q:G
20	0.7012	11.91	10.32	1.320	118	8	6	0.2797	0	131	9	127	9	PDB 3i3f:A	PDB 5v4d:E
21	0.6999	9.529	9.445	1.355	117	7	5	0.2479	0	131	9	124	8	PDB 3i3f:A	PDB 3l7q:F
22	0.6997	9.063	9.183	1.356	117	7	5	0.2479	0	131	9	124	8	PDB 3i3f:A	PDB 3l7q:C
23	0.6996	7.824	8.564	1.357	117	7	5	0.2479	0	131	9	124	8	PDB 3i3f:A	PDB 3l7q:A
24	0.6992	9.063	9.012	1.327	117	8	5	0.3419	0	131	9	125	9	PDB 3i3f:A	PDB 3m4s:E
25	0.6988	9.517	9.401	1.362	117	7	5	0.2479	0	131	9	124	8	PDB 3i3f:A	PDB 3l7q:H
26	0.6984	10.04	9.475	1.461	118	8	4	0.2627	0	131	9	123	9	PDB 3i3f:A	PDB 3k0t:B
27	0.6975	9.841	9.382	1.299	116	8	4	0.2586	0	131	9	124	9	PDB 3i3f:A	PDB 6l8p:C
28	0.6968	12.1	10.4	1.309	117	8	6	0.2821	0	131	9	126	9	PDB 3i3f:A	PDB 5v4d:C
29	0.6967	9.224	9.089	1.341	117	8	5	0.3419	0	131	9	125	9	PDB 3i3f:A	PDB 3m4s:A
30	0.6966	9.381	9.166	1.333	121	8	5	0.2645	0	131	9	134	9	PDB 3i3f:A	PDB 6tcc:A
31	0.6966	9.35	9.151	1.378	118	8	5	0.3305	0	131	9	126	9	PDB 3i3f:A	PDB 3m4s:F
32	0.6966	9.152	9.227	1.374	117	7	5	0.2479	0	131	9	124	8	PDB 3i3f:A	PDB 3l7q:D
33	0.6956	7.826	8.564	1.423	120	7	5	0.2833	0	131	9	129	8	PDB 3i3f:A	PDB 6tcd:B
34	0.6937	8.139	8.765	1.394	118	7	5	0.2881	0	131	9	126	8	PDB 3i3f:A	PDB 6tcd:F
35	0.6919	9.094	9.198	1.401	117	7	5	0.2479	0	131	9	124	8	PDB 3i3f:A	PDB 3l7q:I
36	0.6912	12.17	10.43	1.341	117	8	6	0.2821	0	131	9	126	9	PDB 3i3f:A	PDB 5v4d:F
37	0.6912	9.808	9.367	1.373	117	8	5	0.3333	0	131	9	125	9	PDB 3i3f:A	PDB 3m4s:B
38	0.6911	10.24	9.568	1.374	117	8	5	0.3333	0	131	9	125	9	PDB 3i3f:A	PDB 3m4s:D
39	0.6908	10.07	9.49	1.506	119	8	5	0.2605	0	131	9	125	9	PDB 3i3f:A	PDB 3r0p:A
40	0.6907	10.1	9.506	1.376	117	8	5	0.3333	0	131	9	125	9	PDB 3i3f:A	PDB 3m4s:C
41	0.6906	12.06	10.38	1.381	118	8	6	0.2797	0	131	9	127	9	PDB 3i3f:A	PDB 5v4d:A
42	0.6895	9.264	9.105	1.449	118	8	5	0.2458	0	131	9	125	9	PDB 3i3f:A	PDB 2dyy:A
43	0.6894	8.837	8.905	1.483	119	8	5	0.2605	0	131	9	126	9	PDB 3i3f:A	PDB 3r0p:D
44	0.6894	11.99	10.35	1.388	118	8	6	0.2797	0	131	9	127	9	PDB 3i3f:A	PDB 5v4d:D
45	0.6886	10.34	9.614	1.454	118	8	5	0.2627	0	131	9	125	9	PDB 3i3f:A	PDB 3r0p:E
46	0.6884	9.971	9.444	1.420	117	8	4	0.265	0	131	9	124	9	PDB 3i3f:A	PDB 3k0t:C
47	0.6881	11.77	10.25	1.426	118	8	6	0.2797	0	131	9	126	9	PDB 3i3f:A	PDB 5v4d:B
48	0.6874	6.898	8.12	1.619	122	7	5	0.2131	0	131	9	128	8	PDB 3i3f:A	PDB 3lme:L
49	0.6868	11.77	10.25	1.494	118	8	4	0.2542	0	131	9	124	9	PDB 3i3f:A	PDB 7kgc:C
50	0.6866	8.944	9.125	1.431	117	7	5	0.2479	0	131	9	124	8	PDB 3i3f:A	PDB 3l7q:E

51	0.6854	8.884	8.92	1.505	119	8	5	0.2605	0	131	9	126	9	PDB 3i3f:A	PDB 3r0p:F
52	0.6834	9.17	9.058	1.374	114	8	4	0.2456	0	131	9	120	9	PDB 3i3f:A	PDB 2dyy:E
53	0.6828	12.13	10.41	1.385	116	8	4	0.25	0	131	9	124	9	PDB 3i3f:A	PDB 7kgc:A
54	0.6824	12.22	10.32	1.388	116	8	4	0.25	0	131	9	124	8	PDB 3i3f:A	PDB 7kgc:B
55	0.6816	7.605	8.449	1.510	122	7	5	0.2787	0	131	9	133	8	PDB 3i3f:A	PDB 6tcd:D
56	0.6808	9.042	8.997	1.494	117	8	4	0.265	0	131	9	123	9	PDB 3i3f:A	PDB 3k0t:A
57	0.6789	9.358	9.151	1.412	117	8	6	0.2564	0	131	9	126	9	PDB 3i3f:A	PDB 2dyy:D
58	0.6775	9.651	9.289	1.447	116	8	5	0.2414	0	131	9	123	9	PDB 3i3f:A	PDB 2dyy:K
59	0.6773	7.114	8.235	1.644	122	7	5	0.1967	0	131	9	129	8	PDB 3i3f:A	PDB 3lme:H
60	0.6771	6.924	8.135	1.673	122	7	5	0.1967	0	131	9	128	8	PDB 3i3f:A	PDB 3lme:I
61	0.6769	6.924	8.135	1.647	122	7	5	0.1967	0	131	9	129	8	PDB 3i3f:A	PDB 3lme:K
62	0.6768	10.92	9.878	1.459	118	8	5	0.1949	0	131	9	127	9	PDB 3i3f:A	PDB 2uyj:C
63	0.6765	11.02	9.925	1.395	117	8	5	0.1966	0	131	9	127	9	PDB 3i3f:A	PDB 2uyn:A
64	0.6765	10.99	9.909	1.461	118	8	5	0.1949	0	131	9	127	9	PDB 3i3f:A	PDB 2uyp:A
65	0.6761	8.815	8.866	1.708	123	7	5	0.2033	0	131	9	129	7	PDB 3i3f:A	PDB 3lme:B
66	0.6758	7.562	8.221	1.652	122	7	5	0.1967	0	131	9	129	7	PDB 3i3f:A	PDB 3lme:E
67	0.6758	10.92	9.878	1.399	117	8	5	0.1966	0	131	9	127	9	PDB 3i3f:A	PDB 2uyk:A
68	0.6757	9.192	9.074	1.461	117	8	5	0.265	0	131	9	125	9	PDB 3i3f:A	PDB 3r0p:B
69	0.6754	10.82	9.831	1.467	118	8	5	0.1949	0	131	9	127	9	PDB 3i3f:A	PDB 2uyp:C
70	0.675	10.35	9.475	1.687	123	8	5	0.2358	0	131	9	130	8	PDB 3i3f:A	PDB 3v4d:D
71	0.675	9.224	9.089	1.533	119	8	5	0.2689	0	131	9	127	9	PDB 3i3f:A	PDB 3r0p:C
72	0.6747	6.845	8.092	1.658	122	7	5	0.1967	0	131	9	129	8	PDB 3i3f:A	PDB 3lme:G
73	0.674	10.95	9.894	1.475	118	8	5	0.1949	0	131	9	127	9	PDB 3i3f:A	PDB 2uyn:C
74	0.6739	8.395	8.895	1.662	122	7	5	0.1967	0	131	9	129	8	PDB 3i3f:A	PDB 3lme:J
75	0.6737	10.88	9.863	1.511	119	8	5	0.1933	0	131	9	128	9	PDB 3i3f:A	PDB 2uyj:B
76	0.6735	10.85	9.847	1.478	118	8	5	0.1949	0	131	9	127	9	PDB 3i3f:A	PDB 2uyk:B
77	0.6728	10.99	9.909	1.417	117	8	5	0.1966	0	131	9	127	9	PDB 3i3f:A	PDB 2uyn:B
78	0.6716	10.68	9.816	1.424	117	8	5	0.2137	0	131	9	127	10	PDB 3i3f:A	PDB 3vcz:B
79	0.6715	6.739	8.035	1.729	122	7	4	0.1967	0	131	9	127	8	PDB 3i3f:A	PDB 3lme:C
80	0.6711	10.75	9.8	1.426	117	8	5	0.2137	0	131	9	127	9	PDB 3i3f:A	PDB 3vcz:C
81	0.6704	10.32	9.459	1.615	119	8	4	0.2353	0	131	9	125	8	PDB 3i3f:A	PDB 3v4d:B
82	0.6702	9.455	9.197	1.316	118	8	5	0.3305	0	131	9	133	9	PDB 3i3f:A	PDB 3mqw:A
83	0.6702	10.95	9.894	1.467	118	8	5	0.1949	0	131	9	128	9	PDB 3i3f:A	PDB 2uyk:C
84	0.6698	6.221	7.749	1.738	122	7	5	0.2049	0	131	9	127	8	PDB 3i3f:A	PDB 3lme:F
85	0.6697	9.17	9.058	1.460	116	8	6	0.2672	0	131	9	124	9	PDB 3i3f:A	PDB 2dyy:J
86	0.6694	6.713	8.02	1.686	122	7	6	0.2049	0	131	9	129	8	PDB 3i3f:A	PDB 3lme:D
87	0.6673	10.61	9.738	1.448	117	8	5	0.2137	0	131	9	127	9	PDB 3i3f:A	PDB 3vcz:A
88	0.6659	10.3	9.599	1.554	119	8	5	0.1933	0	131	9	128	9	PDB 3i3f:A	PDB 2uyp:B
89	0.6659	10.69	9.63	1.540	115	8	5	0.2435	0	131	9	120	8	PDB 3i3f:A	PDB 3v4d:A
90	0.6653	10.42	9.506	1.553	118	8	4	0.2373	0	131	9	126	8	PDB 3i3f:A	PDB 3v4d:C
91	0.6651	10.88	9.863	1.496	118	8	5	0.1949	0	131	9	128	9	PDB 3i3f:A	PDB 2uyj:A
92	0.6639	9.327	9.135	1.451	113	8	5	0.2566	0	131	9	119	9	PDB 3i3f:A	PDB 2dyy:L
93	0.6599	6.505	7.906	1.678	121	7	6	0.1983	0	131	9	129	8	PDB 3i3f:A	PDB 3lme:A
94	0.6571	9.735	9.182	1.627	118	8	5	0.2373	0	131	9	125	8	PDB 3i3f:A	PDB 3v4d:E
95	0.6564	9.969	9.135	1.389	111	7	5	0.2523	0	131	9	118	8	PDB 3i3f:A	PDB 2dyy:F
96	0.655	9.318	9.135	1.340	117	8	5	0.3419	0	131	9	133	9	PDB 3i3f:A	PDB 3mqw:F
97	0.6548	9.672	9.151	1.713	123	8	4	0.2276	0	131	9	133	9	PDB 3i3f:A	PDB 3v4d:F
98	0.6522	8.283	8.459	1.372	109	8	6	0.2661	0	131	9	115	8	PDB 3i3f:A	PDB 3k12:C
99	0.652	9.48	9.212	1.337	118	8	5	0.3305	0	131	9	136	9	PDB 3i3f:A	PDB 3mqw:E
100	0.648	9.542	8.935	1.435	110	7	5	0.2455	0	131	9	116	9	PDB 3i3f:A	PDB 2dyy:I
101	0.6473	8.35	8.49	1.402	109	8	6	0.2661	0	131	9	115	8	PDB 3i3f:A	PDB 3k12:E
102	0.6463	9.224	9.089	1.343	118	8	5	0.3305	0	131	9	137	9	PDB 3i3f:A	PDB 3mqw:C
103	0.6453	8.902	8.597	1.530	114	7	4	0.2368	0	131	9	122	8	PDB 3i3f:A	PDB 2dyy:H
104	0.6406	10.65	9.614	1.336	108	8	5	0.213	0	131	9	116	8	PDB 3i3f:A	PDB 3kjk:E
105	0.639	10.62	9.599	1.385	109	8	5	0.211	0	131	9	117	8	PDB 3i3f:A	PDB 3kjj:I
106	0.6386	8.229	8.428	1.344	107	8	5	0.271	0	131	9	114	8	PDB 3i3f:A	PDB 3k12:F

107	0.6374	10.25	9.428	1.390	108	8	5	0.213	0	131	9	115	8	PDB 3i3f:A	PDB 3kjj:L
108	0.635	10.72	9.645	1.337	108	8	5	0.213	0	131	9	117	8	PDB 3i3f:A	PDB 3kjk:L
109	0.6292	9.318	9.135	1.335	118	8	5	0.339	0	131	9	141	9	PDB 3i3f:A	PDB 3mqw:B
110	0.6254	8.952	8.797	1.295	108	8	6	0.2685	0	131	9	120	8	PDB 3i3f:A	PDB 3k12:D
111	0.6252	9.322	9.135	1.333	118	8	5	0.339	0	131	9	142	9	PDB 3i3f:A	PDB 3mqw:D
112	0.6232	8.024	8.49	1.619	112	8	5	0.2589	0	131	9	119	9	PDB 3i3f:A	PDB 2dyy:C
113	0.6212	9.082	8.705	1.565	111	7	5	0.2342	0	131	9	119	9	PDB 3i3f:A	PDB 2dyy:B
114	0.6158	7.784	8.335	1.644	119	7	5	0.2185	0	131	9	135	7	PDB 3i3f:A	PDB 6izh:B
115	0.6149	7.702	8.292	1.649	119	7	5	0.2185	0	131	9	135	7	PDB 3i3f:A	PDB 6izh:H
116	0.614	8.33	8.621	1.485	104	7	3	0.3173	0	131	9	108	7	PDB 3i3f:A	PDB 7cd2:A
117	0.6127	9.103	9.241	1.176	100	7	5	0.24	0	131	9	108	8	PDB 3i3f:A	PDB 5hp8:B
118	0.6117	8.192	8.55	1.499	104	7	3	0.3077	0	131	9	108	7	PDB 3i3f:A	PDB 7cd2:G
119	0.6116	7.948	8.421	1.668	119	7	5	0.2269	0	131	9	135	7	PDB 3i3f:A	PDB 6izh:G
120	0.6113	8.953	9.169	1.271	101	7	5	0.2376	0	131	9	108	8	PDB 3i3f:A	PDB 5hp8:A
121	0.611	8.587	8.751	1.646	119	7	5	0.2185	0	131	9	136	7	PDB 3i3f:A	PDB 6izh:I
122	0.6104	8.219	8.564	1.507	104	7	3	0.3077	0	131	9	108	7	PDB 3i3f:A	PDB 7cd3:A
123	0.6102	8.747	9.068	1.194	100	7	5	0.24	0	131	9	108	8	PDB 3i3f:A	PDB 5hp8:C
124	0.6094	8.389	8.65	1.476	103	7	3	0.3107	0	131	9	107	7	PDB 3i3f:A	PDB 7cd2:Q
125	0.6088	8.842	8.88	1.572	118	7	5	0.2203	0	131	9	137	7	PDB 3i3f:A	PDB 6izh:F
126	0.6087	9.103	9.241	1.204	100	7	5	0.24	0	131	9	108	8	PDB 3i3f:A	PDB 5hp7:A
127	0.6086	7.971	8.435	1.519	104	7	3	0.3077	0	131	9	108	7	PDB 3i3f:A	PDB 7cd2:L
128	0.6084	11.44	9.972	1.760	111	8	7	0.2523	0	131	9	115	8	PDB 3i3f:A	PDB 3gtz:C
129	0.6083	7.807	8.349	1.593	106	7	4	0.3019	0	131	9	110	7	PDB 3i3f:A	PDB 7cd2:J
130	0.6079	8.56	8.597	1.636	109	8	6	0.2569	0	131	9	115	8	PDB 3i3f:A	PDB 3gtz:A
131	0.6054	7.781	8.335	1.686	120	7	4	0.225	0	131	9	138	7	PDB 3i3f:A	PDB 6izh:C
132	0.6046	8.615	8.628	1.589	108	8	6	0.2593	0	131	9	115	8	PDB 3i3f:A	PDB 3gtz:B
133	0.6038	8.387	8.65	1.510	103	7	3	0.3107	0	131	9	107	7	PDB 3i3f:A	PDB 7cd2:E
134	0.602	8.587	8.751	1.672	119	7	5	0.2185	0	131	9	137	7	PDB 3i3f:A	PDB 6izh:D
135	0.6019	6.827	7.82	1.522	103	7	3	0.3107	0	131	9	107	7	PDB 3i3f:A	PDB 7cd2:I
136	0.5996	8.247	8.578	1.573	104	7	3	0.3077	0	131	9	108	7	PDB 3i3f:A	PDB 7cd4:B
137	0.5994	6.541	7.663	1.574	104	7	3	0.3077	0	131	9	108	7	PDB 3i3f:A	PDB 7cd4:D
138	0.5989	6.57	7.677	1.541	103	7	3	0.3107	0	131	9	107	7	PDB 3i3f:A	PDB 7cd2:H
139	0.5979	7.971	8.435	1.547	103	7	3	0.3107	0	131	9	107	7	PDB 3i3f:A	PDB 7cd2:K
140	0.5978	8.36	8.636	1.475	102	7	3	0.3039	0	131	9	107	7	PDB 3i3f:A	PDB 7cd3:B
141	0.5969	8.277	8.593	1.519	103	7	3	0.3107	0	131	9	108	7	PDB 3i3f:A	PDB 7cd2:M
142	0.5967	8.029	8.464	1.556	117	7	6	0.2393	0	131	9	138	7	PDB 3i3f:A	PDB 6izh:A
143	0.5966	6.594	7.691	1.555	103	7	3	0.3107	0	131	9	107	7	PDB 3i3f:A	PDB 7cd2:N
144	0.5945	6.698	7.749	1.568	103	7	3	0.3107	0	131	9	107	7	PDB 3i3f:A	PDB 7cd2:F
145	0.5943	6.673	7.734	1.569	103	7	3	0.3107	0	131	9	107	7	PDB 3i3f:A	PDB 7cd2:T
146	0.5909	6.644	7.72	1.556	103	7	3	0.3107	0	131	9	108	7	PDB 3i3f:A	PDB 7cd2:P
147	0.5898	7.243	8.049	1.566	104	7	5	0.2885	0	131	9	110	7	PDB 3i3f:A	PDB 7cd3:E
148	0.5859	6.198	7.462	1.587	103	7	3	0.3107	0	131	9	108	7	PDB 3i3f:A	PDB 7cd2:D
149	0.5708	6.899	7.863	1.672	108	7	5	0.2963	0	131	9	119	7	PDB 3i3f:A	PDB 7cd2:C
150	0.567	7.995	8.121	1.524	105	7	5	0.2286	0	131	9	118	9	PDB 3i3f:A	PDB 3i7t:A
151	0.5636	6.928	7.877	1.592	107	7	5	0.2897	0	131	9	121	7	PDB 3i3f:A	PDB 7cd4:C
152	0.5559	6.798	7.806	1.772	103	7	4	0.301	0	131	9	108	7	PDB 3i3f:A	PDB 7cd2:S
153	0.5518	8.728	8.823	1.580	107	7	5	0.2897	0	131	9	124	7	PDB 3i3f:A	PDB 7cd4:A
154	0.5332	10.22	9.413	1.732	115	8	7	0.1652	0	131	9	142	10	PDB 3i3f:A	PDB 2ig8:C
155	0.5259	6.954	7.892	1.548	104	7	4	0.2981	0	131	9	124	7	PDB 3i3f:A	PDB 7cd3:D
156	0.5257	10.25	9.428	1.659	113	8	7	0.1681	0	131	9	142	10	PDB 3i3f:A	PDB 2ig8:A
157	0.5193	6.823	7.82	1.523	103	7	5	0.2913	0	131	9	124	7	PDB 3i3f:A	PDB 7cd3:C
158	0.5153	7.366	8.981	1.497	101	7	5	0.2475	0	131	9	121	9	PDB 3i3f:A	PDB 6izh:E
159	0.5149	9.96	9.289	1.707	113	8	8	0.1681	0	131	9	143	10	PDB 3i3f:A	PDB 2ig8:B
160	0.4748	8.702	8.808	1.410	102	7	4	0.1471	0	131	9	137	9	PDB 3i3f:A	PDB 3lyb:B
161	0.4737	8.759	8.837	1.400	101	7	5	0.1485	0	131	9	135	9	PDB 3i3f:A	PDB 3lyb:D
162	0.4653	9.106	9.01	1.421	100	7	4	0.15	0	131	9	134	9	PDB 3i3f:A	PDB 3lyb:C

163	0.4601	9.076	8.995	1.483	101	7	4	0.1485	0	131	9	136	9	PDB 3i3f:A	PDB 3lyb:A
164	0.3777	1.788	4.367	2.796	104	8	9	0.07692	0	131	9	117	8	PDB 3i3f:A	PDB 3kjj:A
165	0.3761	1.897	4.451	2.834	104	8	8	0.07692	0	131	9	116	8	PDB 3i3f:A	PDB 3kjk:B
166	0.3748	1.838	4.4	2.902	105	8	7	0.07619	0	131	9	116	8	PDB 3i3f:A	PDB 3kjk:F
167	0.3747	1.868	4.417	2.902	105	8	7	0.07619	0	131	9	116	8	PDB 3i3f:A	PDB 3kjk:K
168	0.374	1.819	4.384	2.908	105	8	7	0.07619	0	131	9	116	8	PDB 3i3f:A	PDB 3kjk:I
169	0.3734	1.838	4.4	2.856	104	8	8	0.07692	0	131	9	116	8	PDB 3i3f:A	PDB 3kjj:H
170	0.3731	1.879	4.434	2.858	104	8	8	0.07692	0	131	9	116	8	PDB 3i3f:A	PDB 3kjk:H
171	0.3726	1.855	4.417	2.919	105	8	7	0.07619	0	131	9	116	8	PDB 3i3f:A	PDB 3kjk:A
172	0.3721	1.71	4.3	2.904	106	8	8	0.07547	0	131	9	119	8	PDB 3i3f:A	PDB 3kjj:B
173	0.3721	1.782	4.35	2.815	104	8	9	0.07692	0	131	9	118	8	PDB 3i3f:A	PDB 3kjj:D
174	0.372	1.947	4.484	2.841	104	8	8	0.06731	0	131	9	117	8	PDB 3i3f:A	PDB 3kjk:D
175	0.372	1.849	4.4	2.809	103	8	9	0.07767	0	131	9	116	8	PDB 3i3f:A	PDB 3kjk:C
176	0.3719	1.71	4.3	2.842	104	8	9	0.07692	0	131	9	117	8	PDB 3i3f:A	PDB 3kjj:F
177	0.3713	1.782	4.35	2.930	105	8	7	0.07619	0	131	9	116	8	PDB 3i3f:A	PDB 3kjk:G
178	0.3702	1.819	4.384	2.824	103	8	9	0.07767	0	131	9	116	8	PDB 3i3f:A	PDB 3kjj:E
179	0.3691	1.819	4.384	2.871	105	8	9	0.07619	0	131	9	119	8	PDB 3i3f:A	PDB 3kjj:J
180	0.3654	1.897	4.451	2.844	104	8	10	0.06731	0	131	9	119	8	PDB 3i3f:A	PDB 3kjj:K
181	0.3595	2.483	4.899	2.835	103	8	9	0.07767	0	131	9	119	8	PDB 3i3f:A	PDB 3kjj:C
182	0.3554	1.675	4.266	2.913	102	8	9	0.07843	0	131	9	115	8	PDB 3i3f:A	PDB 3kjj:G
183	0.3469	2.044	4.568	2.781	99	8	10	0.08081	0	131	9	116	8	PDB 3i3f:A	PDB 3kjk:J
184	0.3424	3.462	5.794	2.716	111	7	7	0.1441	0	131	9	151	9	PDB 3i3f:A	PDB 2otm:C
185	0.3344	2.015	5.364	2.712	110	7	6	0.1455	0	131	9	152	10	PDB 3i3f:A	PDB 2otm:A
186	0.3152	4.008	6.103	2.699	109	7	5	0.1651	0	131	9	159	8	PDB 3i3f:A	PDB 3d01:I
187	0.315	4.342	6.322	2.571	106	7	6	0.1604	0	131	9	157	8	PDB 3i3f:A	PDB 3d01:B
188	0.3107	3.993	6.088	2.726	108	7	5	0.1667	0	131	9	157	8	PDB 3i3f:A	PDB 3d01:J
189	0.3104	4.008	6.103	2.729	108	7	5	0.1667	0	131	9	157	8	PDB 3i3f:A	PDB 3d01:H
190	0.3102	3.925	6.044	2.731	108	7	6	0.1574	0	131	9	157	8	PDB 3i3f:A	PDB 3d01:C
191	0.309	4.187	6.22	2.668	106	7	6	0.1698	0	131	9	155	8	PDB 3i3f:A	PDB 3d01:E
192	0.308	4.146	6.191	2.619	106	7	6	0.1698	0	131	9	158	8	PDB 3i3f:A	PDB 3d01:F
193	0.3052	3.925	6.044	2.778	109	7	6	0.156	0	131	9	160	8	PDB 3i3f:A	PDB 3d01:L
194	0.3015	4.288	6.366	2.549	103	7	6	0.165	0	131	9	156	9	PDB 3i3f:A	PDB 3d01:D
195	0.3007	4.167	6.205	2.654	105	7	6	0.1714	0	131	9	157	8	PDB 3i3f:A	PDB 3d01:K
196	0.2994	4.288	6.366	2.550	103	7	6	0.165	0	131	9	157	9	PDB 3i3f:A	PDB 3d01:G
197	0.2973	4.278	6.366	2.553	103	7	6	0.165	0	131	9	158	9	PDB 3i3f:A	PDB 3d01:A
198	0.7244	9.971	9.444	1.356	120	8	4	0.2917	0	131	9	126	9	PDB 3i3f:A	PDB 1jd1:C
199	0.7228	9.805	9.367	1.329	119	8	4	0.2941	0	131	9	125	9	PDB 3i3f:A	PDB 1jd1:F
200	0.7218	9.87	9.397	1.371	120	8	4	0.2917	0	131	9	126	9	PDB 3i3f:A	PDB 1jd1:A
201	0.7214	12.62	10.62	1.302	118	8	4	0.2797	0	131	9	124	9	PDB 3i3f:A	PDB 1qd9:B
202	0.7213	12.84	10.72	1.302	118	8	4	0.2797	0	131	9	124	9	PDB 3i3f:A	PDB 1qd9:A
203	0.7172	12.69	10.65	1.325	118	8	4	0.2797	0	131	9	124	9	PDB 3i3f:A	PDB 1qd9:C
204	0.714	11.06	9.941	1.410	119	8	4	0.2689	0	131	9	124	9	PDB 3i3f:A	PDB 2cvl:A
205	0.7091	10.3	9.599	1.562	121	8	5	0.2727	0	131	9	124	9	PDB 3i3f:A	PDB 2cw4:A
206	0.7088	10.75	9.8	1.438	119	8	4	0.2689	0	131	9	124	9	PDB 3i3f:A	PDB 2cvl:C
207	0.7086	10.78	9.816	1.440	119	8	4	0.2689	0	131	9	124	9	PDB 3i3f:A	PDB 2cvl:D
208	0.7082	10.85	9.847	1.442	119	8	4	0.2689	0	131	9	124	9	PDB 3i3f:A	PDB 2cvl:B
209	0.7077	10.04	9.475	1.447	120	8	5	0.2417	0	131	9	126	9	PDB 3i3f:A	PDB 1x25:A
210	0.7065	10.95	9.894	1.385	118	8	4	0.2797	0	131	9	124	9	PDB 3i3f:A	PDB 2cvl:E
211	0.7047	8.164	8.613	1.353	122	8	5	0.2295	0	131	9	134	10	PDB 3i3f:A	PDB 1oni:F
212	0.7044	10.68	9.769	1.462	119	8	4	0.2773	0	131	9	124	9	PDB 3i3f:A	PDB 2cvl:F
213	0.7043	10.51	9.692	1.362	117	8	4	0.2906	0	131	9	123	9	PDB 3i3f:A	PDB 1jd1:E
214	0.7042	10	9.459	1.405	120	8	5	0.25	0	131	9	128	9	PDB 3i3f:A	PDB 1x25:B
215	0.7032	9.74	9.336	1.372	118	8	4	0.2966	0	131	9	125	9	PDB 3i3f:A	PDB 1jd1:B
216	0.7019	10.57	9.723	1.476	119	8	4	0.2689	0	131	9	124	9	PDB 3i3f:A	PDB 2csl:A
217	0.7002	10.68	9.769	1.485	119	8	4	0.2689	0	131	9	124	9	PDB 3i3f:A	PDB 2csl:B
218	0.6996	8.206	8.582	1.445	123	8	5	0.2276	0	131	9	134	9	PDB 3i3f:A	PDB 1oni:C

219	0.6995	10.61	9.738	1.489	119	8	4	0.2689	0	131	9	124	9	PDB 3i3f:A	PDB 2csl:F
220	0.6994	9.576	9.259	1.358	117	8	4	0.2991	0	131	9	124	9	PDB 3i3f:A	PDB 1jd1:D
221	0.6993	8.558	8.766	1.353	122	8	5	0.2295	0	131	9	135	9	PDB 3i3f:A	PDB 1oni:I
222	0.6992	10.75	9.8	1.490	119	8	4	0.2689	0	131	9	124	9	PDB 3i3f:A	PDB 2csl:D
223	0.6988	8.059	8.505	1.385	122	8	5	0.2295	0	131	9	134	10	PDB 3i3f:A	PDB 1oni:D
224	0.6982	8.481	8.938	1.496	119	7	4	0.2689	0	131	9	124	8	PDB 3i3f:A	PDB 2csl:E
225	0.698	10.58	9.723	1.497	119	8	4	0.2689	0	131	9	124	9	PDB 3i3f:A	PDB 2csl:C
226	0.6974	8.842	8.905	1.393	122	7	5	0.2295	0	131	9	134	10	PDB 3i3f:A	PDB 1oni:B
227	0.6939	8.781	8.874	1.413	122	7	5	0.2295	0	131	9	134	10	PDB 3i3f:A	PDB 1oni:G
228	0.6938	8.33	8.643	1.385	122	8	5	0.2295	0	131	9	135	10	PDB 3i3f:A	PDB 1oni:H
229	0.6932	8.654	8.812	1.359	122	8	5	0.2295	0	131	9	136	9	PDB 3i3f:A	PDB 1oni:A
230	0.6927	8.105	8.582	1.420	122	7	5	0.2295	0	131	9	134	10	PDB 3i3f:A	PDB 1oni:E
231	0.6926	7.96	8.459	1.385	121	7	5	0.2231	0	131	9	133	9	PDB 3i3f:A	PDB 1nq3:F
232	0.6922	8.177	8.567	1.388	121	7	5	0.2231	0	131	9	133	10	PDB 3i3f:A	PDB 1nq3:B
233	0.6888	9.073	9.012	1.436	121	8	5	0.2231	0	131	9	132	9	PDB 3i3f:A	PDB 1qah:A
234	0.6876	11.99	10.35	1.393	117	8	5	0.2906	0	131	9	125	9	PDB 3i3f:A	PDB 1xrg:A
235	0.6843	7.966	8.459	1.433	121	7	5	0.2314	0	131	9	133	10	PDB 3i3f:A	PDB 1nq3:A
236	0.6835	7.995	8.475	1.437	121	7	6	0.2231	0	131	9	133	9	PDB 3i3f:A	PDB 1nq3:C
237	0.6834	10.17	9.537	1.486	119	8	5	0.2605	0	131	9	127	9	PDB 3i3f:A	PDB 2b33:A
238	0.6833	9.552	9.243	1.438	121	7	6	0.2231	0	131	9	133	9	PDB 3i3f:A	PDB 1nq3:E
239	0.681	8.792	8.874	1.480	121	8	5	0.2231	0	131	9	132	9	PDB 3i3f:A	PDB 1qah:B
240	0.6803	10.1	9.506	1.470	118	8	5	0.2627	0	131	9	126	9	PDB 3i3f:A	PDB 2b33:B
241	0.6797	7.873	8.413	1.423	120	7	6	0.225	0	131	9	132	9	PDB 3i3f:A	PDB 1nq3:D
242	0.6706	10.75	9.8	1.429	117	8	5	0.2051	0	131	9	127	9	PDB 3i3f:A	PDB 1qu9:A
243	0.6706	10.75	9.8	1.429	117	8	5	0.2051	0	131	9	127	9	PDB 3i3f:A	PDB 1qu9:B
244	0.6706	10.75	9.8	1.429	117	8	5	0.2051	0	131	9	127	9	PDB 3i3f:A	PDB 1qu9:C
245	0.6516	10.24	9.8	1.443	120	8	5	0.2917	0	131	9	137	10	PDB 3i3f:A	PDB 1xrg:C
246	0.6487	5.621	7.376	1.566	112	7	5	0.2768	0	131	9	116	10	PDB 3i3f:A	PDB 2cwj:A
247	0.6447	9.735	9.568	1.456	120	8	5	0.2917	0	131	9	138	10	PDB 3i3f:A	PDB 1xrg:B
248	0.6357	9.674	9.151	1.665	119	8	6	0.1849	0	131	9	130	8	PDB 3i3f:A	PDB 1pf5:A
249	0.5625	6.931	7.736	1.599	107	8	4	0.2056	0	131	9	121	8	PDB 3i3f:A	PDB 2ewc:K
250	0.5595	6.85	7.69	1.610	106	8	4	0.2075	0	131	9	119	8	PDB 3i3f:A	PDB 2ewc:D
251	0.5575	6.898	7.721	1.594	106	8	4	0.2075	0	131	9	120	8	PDB 3i3f:A	PDB 2ewc:H
252	0.5566	6.877	7.705	1.599	106	8	4	0.2075	0	131	9	120	8	PDB 3i3f:A	PDB 2ewc:I
253	0.5565	6.79	7.659	1.600	106	8	4	0.2075	0	131	9	120	8	PDB 3i3f:A	PDB 2ewc:E
254	0.5563	6.824	7.675	1.601	106	8	4	0.2075	0	131	9	120	8	PDB 3i3f:A	PDB 2ewc:C
255	0.555	6.964	7.752	1.609	106	8	4	0.2075	0	131	9	120	8	PDB 3i3f:A	PDB 2ewc:J
256	0.5543	6.824	7.675	1.614	106	8	4	0.2075	0	131	9	120	8	PDB 3i3f:A	PDB 2ewc:A
257	0.5536	6.904	7.721	1.619	106	8	4	0.2075	0	131	9	120	8	PDB 3i3f:A	PDB 2ewc:G
258	0.5535	6.904	7.721	1.619	106	8	4	0.2075	0	131	9	120	8	PDB 3i3f:A	PDB 2ewc:L
259	0.5497	6.71	7.613	1.652	107	8	4	0.2056	0	131	9	122	8	PDB 3i3f:A	PDB 2ewc:B
260	0.5408	6.71	7.613	1.643	106	8	4	0.2075	0	131	9	122	8	PDB 3i3f:A	PDB 2ewc:F
